# Poly ADP ribosylation and extracellular vesicle activity in rod photoreceptor degeneration

**DOI:** 10.1038/s41598-019-40215-3

**Published:** 2019-03-06

**Authors:** Lorena Vidal-Gil, Javier Sancho-Pelluz, Eberhart Zrenner, Maria Oltra, Ayse Sahaboglu

**Affiliations:** 1 Division of Experimental Ophthalmology, Institute for Ophthalmic Research, Tuebingen, Germany; 2 Escuela de doctorado, Universidad Católica de Valencia San Vicente Mártir, Valencia, Spain; 3 Neurobiología y Neurofisiología, Facultad de Medicina y Odontología, Universidad Católica de Valencia San Vicente Mártir, Valencia, Spain

## Abstract

Retinitis Pigmentosa is a group of inherited neurodegenerative diseases that result in selective cell death of photoreceptors. In the developed world, RP is regarded as the main cause of blindness among the working age population. The precise mechanisms eventually leading to cell death remain unknown and to date no adequate treatment for RP is available. Poly ADP ribose polymerase (PARP) over activity is involved in photoreceptor degeneration and pharmacological inhibition or genetic knock-down PARP1 activity protect photoreceptors in mice models, the mechanism of neuroprotection is not clear yet. Our result indicated that olaparib, a PARP1 inhibitor, significantly rescued photoreceptor cells in rd10 retina. Extracellular vesicles (EVs) were previously recognized as a mechanism for discharging useless cellular components. Growing evidence has elucidated their roles in cell–cell communication by carrying nucleic acids, proteins and lipids that can, in turn, regulate behavior of the target cells. Recent research suggested that EVs extensively participate in progression of diverse blinding diseases, such as age-related macular (AMD) degeneration. Our study demonstrates the involvement of EVs activity in the process of photoreceptor degeneration in a PDE6 mutation. PARP inhibition protects photoreceptors via regulation of the EVs activity in rod photoreceptor degeneration in a PDE6b mutation.

## Introduction

Retinitis pigmentosa (RP) is a group of hereditary retinal degenerative diseases in which rod photoreceptors die due to a genetic mutation, whereas cone photoreceptors disappear secondarily, once rods are gone. While the initial disease symptoms (*i.e*. night blindness) are comparatively mild, the secondary loss of cones ultimately leads to complete blindness. The disease affects approximately 1 in 3,000 to 7,000 people among the working age population in the developed world^1^ and is characterized by strong genetic heterogeneity with causative mutations in more than 65 genes. In 4–8% of human RP cases, the disease is caused by mutations in genes encoding for cGMP specific phosphodiesterase 6 (PDE6)^2,3^. The non-functional enzyme fails to hydrolyze cGMP, causing its accumulation^2,4^. Animal models like the retinal degeneration 1 (*rd1*) and the *rd10* mouse, which harbor a mutated *Pde6b* gene^5–7^, have advanced the understanding of the cellular processes underlying retinal degeneration. Notably, elevated cGMP levels in dying photoreceptors were found to correlate with increased activity of PARP^8,9^. Over activation of PARP was involved in photoreceptor degeneration in different animal models including *rd10* mice model^8^.

Poly-ADP-ribose metabolism is a post-translational modification involved in many cellular pathways such as transcription, DNA repair, and cell death^10^. There are at least 17 different PARP isoforms. Among them, PARP1–116 kDa protein – has become the major focus of research due to its multi-faceted roles in many cellular activities^11,12^. DNA damage by mild genomic stress activates PARP1 whereas massive DNA disruption in several diseases causes excessive PARP1 activation which leads to cell death^13,14^. Excessive activation of PARP1 may lead to excessive utilization of nicotinamide adenine dinucleotide (NAD^+^). Restoration of decreased NAD^+^ requires two or four molecules of adenosine-5′-triphosphate (ATP). Consequently, cellular ATP levels become depleted, leading to an energetic collapse, cellular dysfunction, and eventually cell death^10,15^. PARP is a key factor in a novel form of cell death, which involves accumulation of poly (ADP-ribose) (PAR) and nuclear translocation of apoptosis-inducing factor (AIF) from mitochondria^15^. This PARP-dependent cell death mechanism is tentatively termed *PARthanatos*^10^. In response to DNA damage, PARP1 covalently attaches oligo or poly (ADP ribose) chains on to various acceptor proteins such as histones, DNA polymerases, topoisomerases, and transcription factors or PARP1 itself by transfer of ADP-ribose units from NAD^+^ ^11,13,16^.

Extracellular vesicles (EVs), which are released from many types of cells^17,18^ are widely present in body fluids, including plasma, urine, saliva, pleural and pericardial effusions, and cerebrospinal fluid^19–23^. Previous studies demonstrated that EVs contain various bioactive molecules, such as nucleic acids (DNA, RNA, and miRNA), proteins and lipids and, thus, can be involved in proximal and distal intercellular communication^24^. So far, EVs have been shown to influence immune modulation^25^, tumor invasion^26^, regeneration, and degenerative processes^27^, under both physiological and pathological conditions^28^. EVs have been also found in aqueous humor^29^, and seem to be crucial in cellular communication between retinal cells^30^. Recently, it has been noticed that retinal pigment epithelium (RPE) cells release EVs, and that the vesicle number and cargo can vary depending on cell homeostasis^31,32^. Additionally, it has been observed that certain retinal EVs are able to promote or inhibit neovascularization in different systems^33^, including the retina^34–36^.

Although it is known that PARP^8^ and EV activity^34^ take role in retinal degenerative diseases, no information is available for the link of PARP and EV activity in neurodegeneration and neuroprotection of the retina. Here we tested whether EVs are involved in inherited retinal degeneration and if there is a connection between PARP and EV activity in degeneration and neuroprotection of photoreceptors in RP. We show that the expression of retinal EVs changes in rod photoreceptor degeneration. Our results provide novel insight on how PARP inhibition protects photoreceptors and identifies for the first time, the link between PARP activity and EV release in photoreceptor degeneration.

## Results

### PARP inhibition with olaparib rescues photoreceptors in *rd10* retinal explant cultures

**Figure 1:**
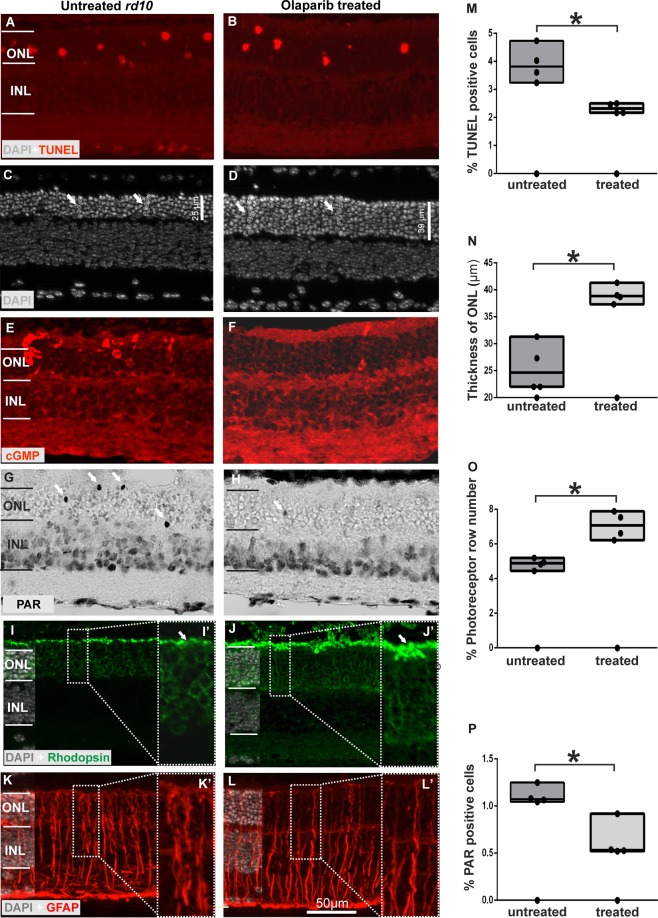
PARP inhibition protects photoreceptor degeneration and changes rhodopsin, PARylation, GFAP level in *rd10* retina. TUNEL assay for dying cells indicated significantly decreased numbers of positive cells (**A,B,M**). The photoreceptor row numbers and the thickness of ONL (**C,D**) increased for 100 nM olaparip treated groups (**N,O**). Similar to TUNEL, immunohistochemical analysis of PARylation in photoreceptors (see errors) revealed significantly decreased numbers of PAR positive cells for 100 nM olaparib treated groups (**G,H,P**). In addition, cGMP staining showed decreased cGMP level in treated groups (**E,F**). The rhodopsin expression increased in olaparib treated groups (see errors) (**I,J**). GFAP staining to observe Muller cell activity showed less GFAP expression for treated group (**K,L**). The images shown are representative for observations on at least three different specimens for each genotype/treatment condition. N ≥ 4, significance levels: ^*^P < 0.05.

Previous studies showed that 100 nM olaparib, a PARP inhibitor, is the most effective concentration to protect photoreceptors in the PDE6 beta mutant, *rd1* murine model^37^. Similarly, olaparib exhibited neuroprotective effect on another PDE6 beta mutant, the *rd10* mouse, with a significant reduction of TUNEL positive cells at 100 nM olaparib (untreated: 3.82 n = 4; treated: 2.31 n = 4; p < 0.1, Fig. 1A,B,M). Moreover, the number of photoreceptor rows and the thickness of ONL increased significantly when the cultures were treated with 100 nM olaparib (photoreceptor rows untreated: 4.8 ± 0.15 SEM, n = 4, treated: 7.1 ± 0.38 SEM, n = 4; p < 0.01, thickness of ONL untreated: 25.6 µm ± 2.2 SEM, n = 4, treated: 39 µm ± 0.8 SEM, n = 5; p < 0.01, Fig. 1C,D,N,O).

Furthermore, we observed that the level of cGMP was reduced when 100 nM olaparib was added to the cultures (Fig. 1E,F), confirming previous studies^9,37^. The effectiveness of PARP inhibition by olaparib was analyzed by staining for PARylated proteins in photoreceptors. The quantification of PAR positive cells in outer nuclear layer (ONL) indicated a significant decrease of PAR positivity for the 100 nM olaparib treated group (untreated: 1.11 ± 0.05 SEM, n = 4; treated: 0.62 ± 0.09 SEM, n = 4; p < 0.01, Fig. 1G,H,P).

### PARP inhibition improves rod outer segment development and Müller cell activity in *rd10* retinal cultures

To test whether and how PARP inhibition would affect rod photoreceptor morphology we performed immunostainings targeting rhodopsin. At P18, wild-type retina illustrated the normal development of rod outer segments (ROS), characterized by strong rhodopsin immunoreactivity *in vivo*. In *rd10*, ROS rhodopsin expression was significantly reduced when compared to *wt* (Supplementary Fig. 1A,B). Likewise, *rd10* explant cultures showed a low expression of rhodopsin at P18 *in vitro* (Fig. 1I). The findings from organ culture confirm previous studies^38^ where *rd10* explant cultures treated with 100 nM olaparib showed significant OS growth with augmented rhodopsin immunoreactivity (Fig. 1I,I’ and J,J’), indicating that the treatment had in part restored OS architecture.

We also analyzed the activity of Müller cells by glial fibrillary acidic protein (GFAP). Although, GFAP-positive fibers span the retina from the inner limiting membrane to the external limiting membrane in untreated *rd10* retinae, olaparib-treated explants showed a reduction in the expression of GFAP in the ONL (Fig. 1K,K’ and L,L’).

### Characterisation of EV activity in *rd10* retina

**Figure 2:**
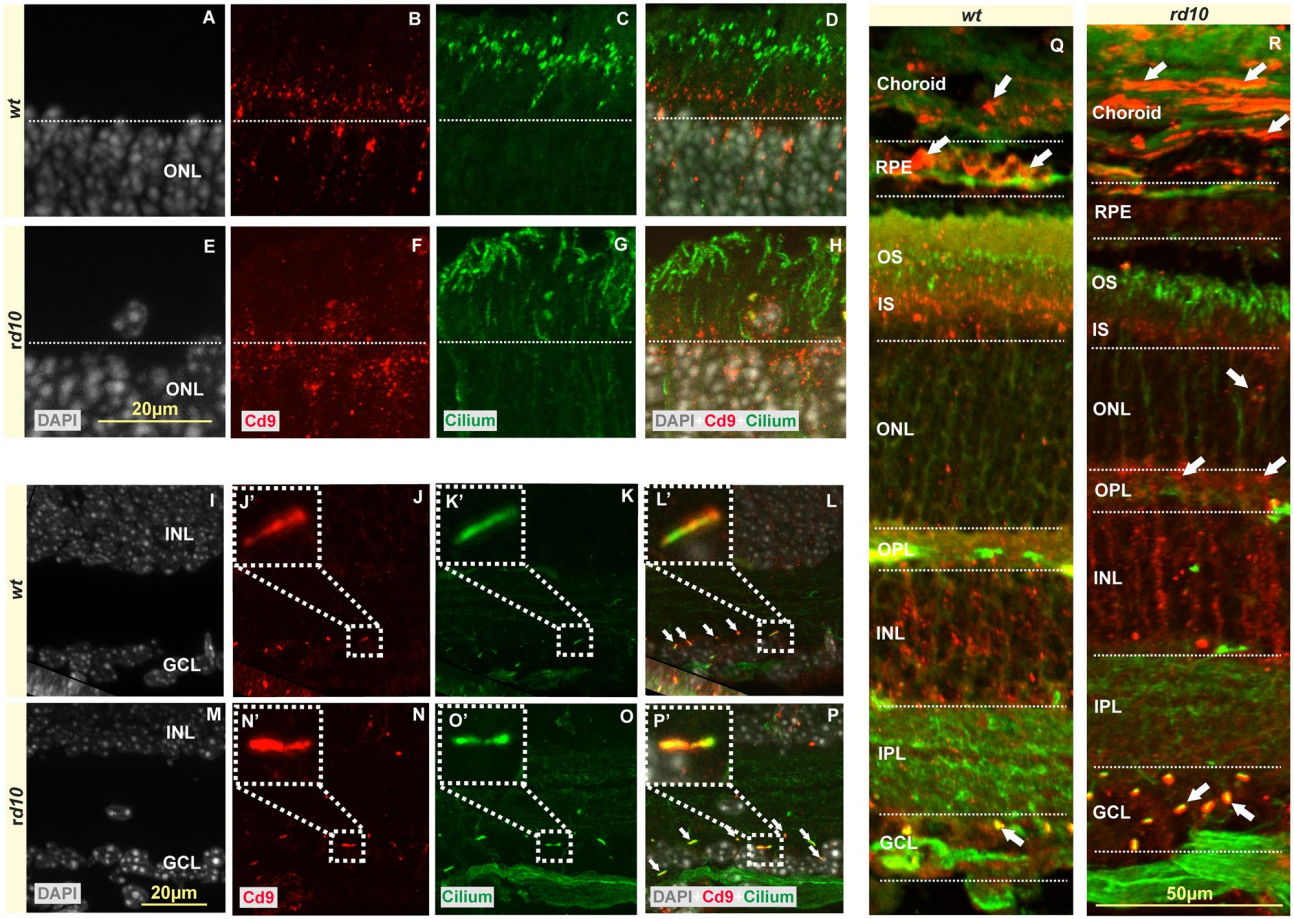
EVs and cilium in *rd10* and corresponding *wt* retinae. The expression of CD9 showed that EVs were placed at choroid, RPE, inner segments, INL, GCL (see errors) of *rd10* and *wt* retinae at P18 (**A–S**). The EVs in the GCL were colocalized with cilium, although EVs and ciliums were observed in the inner and outer segments *rd10* and *wt* retinae respectively (**I–S**). The images shown are representative for observations on at least three different specimens for each genotype/treatment condition.

**Figure 3:**
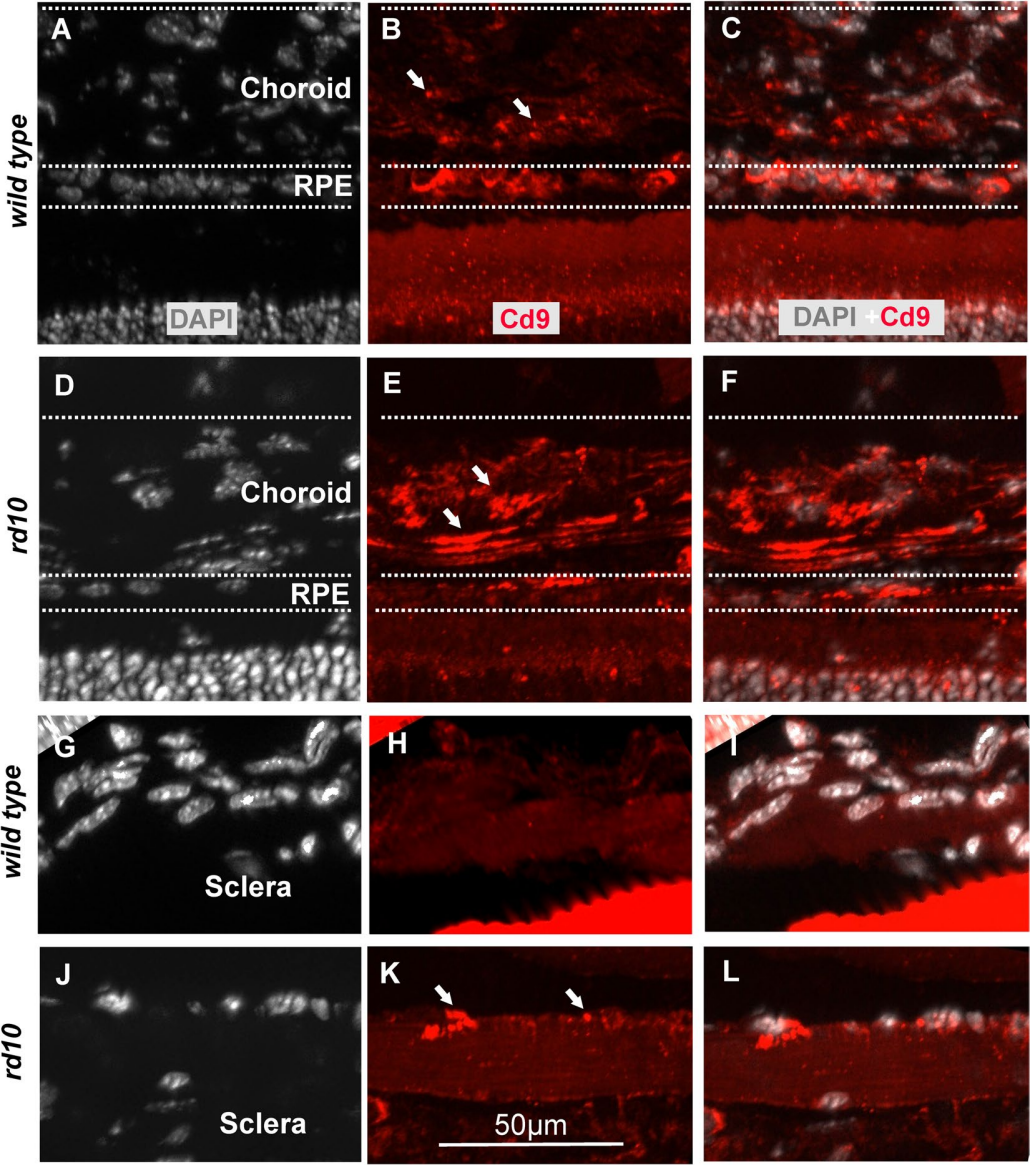
Expression of CD9 in choroid and sclera of *rd10* and *wt ex vivo* retinae at P18. CD9 expression showed strong immunoreactivity for choroid of *rd10* retinae (**A**) compared to corresponding *wt* at P18. There was hardly any CD9 expression for sclera of *wt* and on the other hand, there was CD9 expression localised with the nuclei of sclera of *rd10* mice (**B**).

CD9 is a protein of the tetraspanin family that has been observed in cell membranes and in the surface of EVs^39–41^. We analysed the location of CD9 expression in *rd10* mice retinae and corresponding *wt in vivo* at P18. The EVs were observed throughout the *rd10* and *wt* retinae, RPE, and choroid (Fig. 2A–R). CD9 expression was observed at choroid, retinal pigment epithelium (RPE), inner segment (IS), inner nuclear layer (INL), and ganglion cell layer of *w*t mice eye at P18 (Figs 2Q and 3). In *rd10* mice, CD9 expression was highly increased in choroid, ONL, and INL, some photoreceptor nuclei (see error, Figs 2R and 3) and decreased in RPE layer when compared to corresponding *wt* mice (Figs 2A–R and 3).

The localization of EVs was analysed by colabelling with cilium, by using an antibody against polyglutamylation modification. It has been already shown that cilia appear to interact purposefully with cells and EVs from their surrounding cellular neighborhood^42^. Although the EVs in inner segment of retina took place next to cilium, the EVs in ganglion cell layer colocalised with cilium completely (Fig. 2I–P).

### PARP inhibition affects EV release in photoreceptor *degeneration*

**Figure 4:**
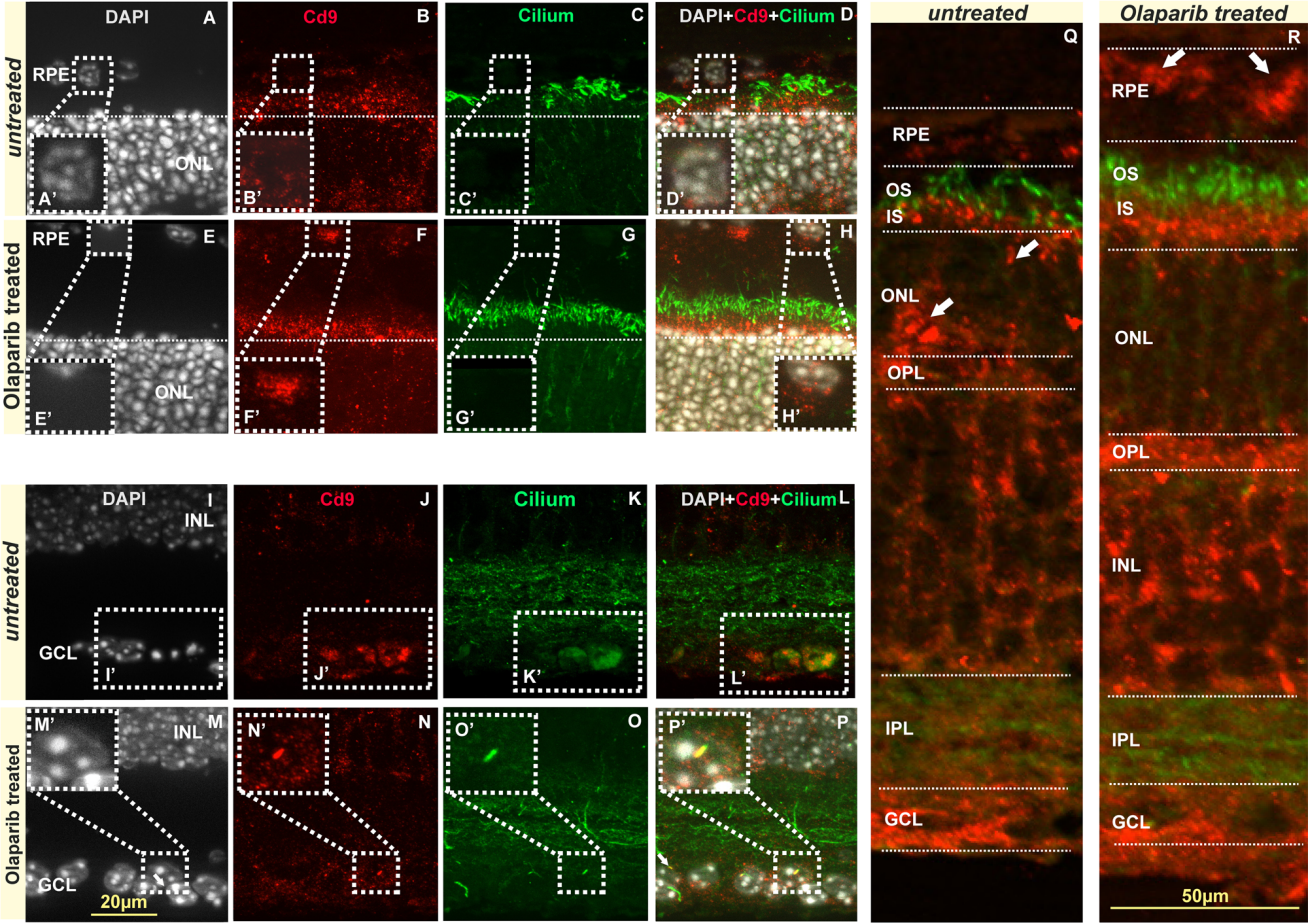
EVs and cilium in untreated and olaparib treated *rd10* retinae. The EVs in the GCL were colocalized with cilium, although EVs and ciliums were observed in the inner and outer segments *rd10* and *wt* retinae respectively (**I–S**). The images shown are representative for observations on at least three different specimens for each genotype/treatment condition.

When *rd10* retinal explants were treated with PARP inhibitor olaparib, some changes were observed in the release of EVs from retinal cells. According to data collected by immunofluorescence, CD9 expression was apparently enhanced in RPE cells of *rd10* after PARP inhibition (Fig. 4A–H). Nevertheless, CD9 expression in the GCL was lower after olaparib treatment (Fig. 4I–P). Moreover, CD9 reactivity was reduced in the ONL and inner segments of *rd10* retinas after olaparib treatment (3q-r).

**Figure 5:**
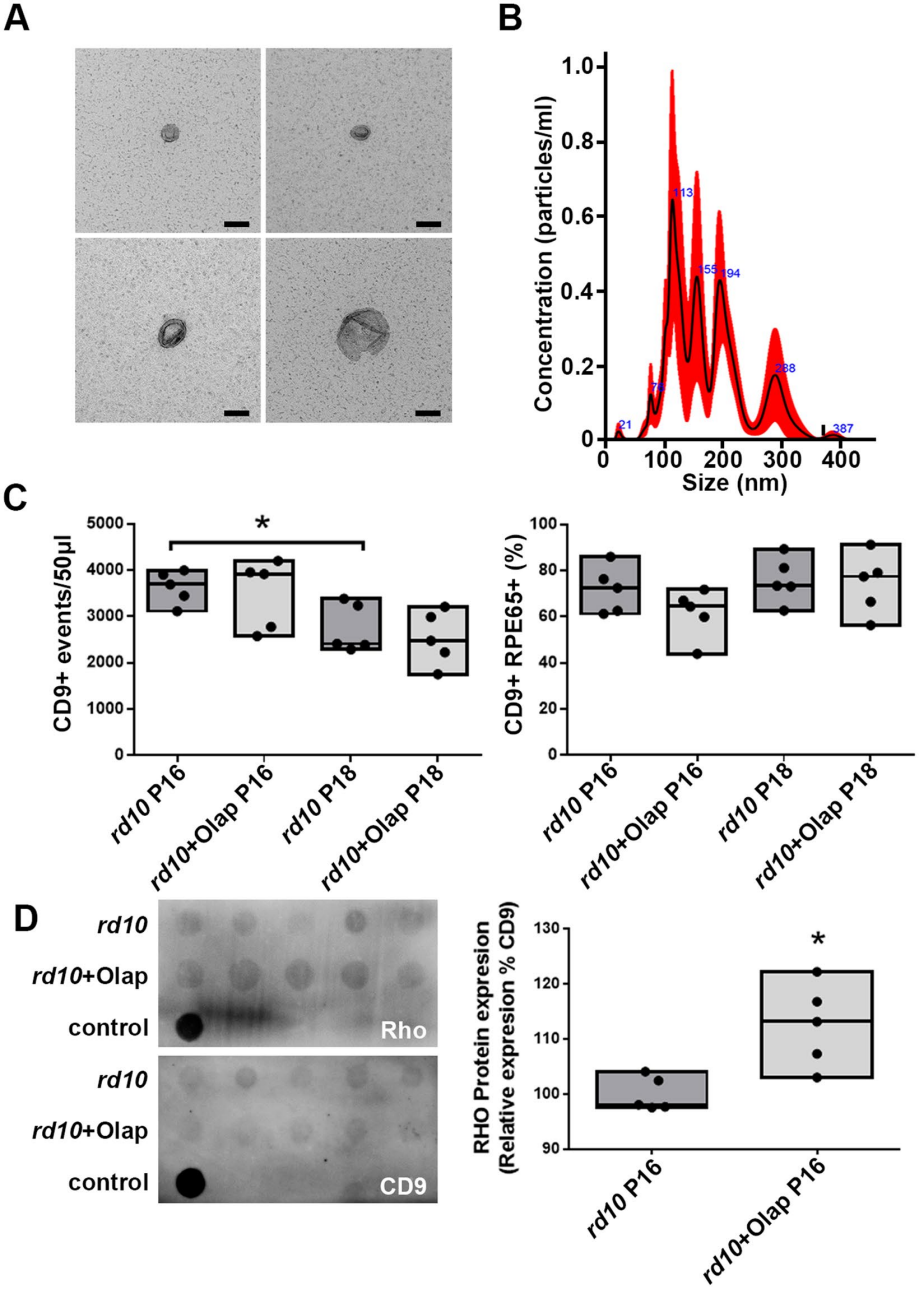
EVs from retinal cells. EVs of different size were observed under the electron microscope (**A**). Size of the EVs was confirmed by Nanosight, a tracking system based in the Brownian movement of the particles (**B**). EVs were quantified by means of flow cytometry, targeting CD9, a well-known marker for them (**C**). Significant differences in the release of EVs were observed between *rd10* retinas at P16 and P18, where the latest showed less amount. Conversely, the ratio of RPE65 protein in EVs did not seem to change. Presence of rhodopsin in retinal EVs was studied using dot blot (**D**). Rhodopsin seemed to be present in EVs, being enhanced when *rd10* retinas were treated with olaparib (bar diagram in **D**). Values are expressed as mean ± SEM (N ≥ 5). Significance levels: ^*^P < 0.05 and ^**^P < 0.01, scale bar 100 nm.

Moreover, cultured mediums from *rd10* retinal explants, treated and untreated with olaparib, were collected to analyze their composition of EVs. Subsequently, EVs were isolated as explained above, and they were observed under the electron microscope. Even though, most of the EVs observed matched morphology and the size of EVs (Fig. 5A, first three panels), we found a few which were oversized, being probably microvesicles (Fig. 5A, last panel). Size of particles was double-checked observing their Brownian motion using a particle size track system (Nanosight). Most of the particles found sized around 100 nm, but there were also bigger vesicles, which matched what was observed previously (Fig. 5B).

The nature of the vesicles was also studied by means of flow cytometry and dot blot. Isolated EVs from untreated and treated cultures were analyzed by flow cytometry using different antibodies: CD9 and RPE65. Events positive for CD9 were counted by the cytometer and there was not a significant change between samples. Thus, total number of EVs was similar before and after treatment (Fig. 5C). Moreover, RPE65-positive events matching with CD9 (potentially attached to RPE-released EVs), did not present a significant difference.

Rhodopsin expression in EVs was analyzed by means of dot blot (Fig. 5D). It was noticed for the first time that EVs from retinal cells – most probably from rod-shaped photoreceptors – express rhodopsin. EVs from untreated *rd10* retinas contained less amount of rhodopsin than those which photoreceptors were rescued by using the PARP inhibitor olaparib. These results are consistent with the observations made by immunohistochemical analysis.

## Discussion

In the last 10 years, it was shown that PARP over-activity is involved in retinal degeneration in different animal models of retinal degeneration. The peak of PARP activity matched at the peak of photoreceptor cell death or before the cell death for some mice models, such as *rd2* and PDE6a models^8,38,43^. In addition, it was noticed that PARP inhibition or knocking out of PARP1 protected photoreceptor cells in *rd1*, *rd2* and other PDE6a mice models^37,38,43^. In this study, our findings are consistent with *previous* ones^37,38,43^ that PARP inhibition significantly decreased PARylated proteins and cGMP level in photoreceptor layer in *rd10* model. Although, the role of excessive PARP activity in photoreceptor cell death is known, the exact mechanism lying back is unknown. In addition, how PARP inhibition protects photoreceptor cells is still under debate. Our present work illustrates the link between PARP and EV release in retinal degeneration in *rd10* mice. We also present the connection of cilium and EV activity highlighting the possible pathway covering PARP-EV and cilium. Further investigations on the molecular mechanism of PARP dependent cell death in retinal degeneration could bring new targets to prevent photoreceptor degeneration, eventually hereditary retinal dystrophies.

Previous data indicate that RPE cells undergoing oxidative stress (OS) release higher number of EVs to the medium and the cargo of these vesicles was significantly modified, impacting neighboring tissues, such as the choroids and photoreceptor cells^30,34,44^. It is also known that the epithelium, under OS, enhances autophagic mechanisms^45^. Moreover, these two mechanisms, autophagy and EV release, seem to share a common pathway^35^. Although there are important efforts to study EVs released from photoreceptors^46^, the truth is that most of the studies that analyze retinal EVs have been focused in the RPE^31,44^. In the present study we observed how the total number of EVs remains unaffected, when retinas from control and mutant animals were cultured. Moreover, the total number of EVs did not change when olaparib – a PARP-inhibiting drug that prolongs the life of photoreceptors – was added to the medium. Nevertheless, we also noticed that expression of CD9 was altered if independent areas of the retina were studied. In the ONL of *rd10* animals, CD9 expression is higher than in *wt* controls. This data matches previous observations: any stress in the cells will increase the number of vesicles released^34,47^. However, when olaparib was added, CD9 expression seem to decrease in the ONL of *rd10*. Conversely, RPE cells tended to release more EVs in *wt* than in *rd10*. After olaparib treatment, RPE-derived EVs where comparable to the untreated condition.

Cilia are found in many cells and play important roles in development, differentiation and function of many tissues. Cilia have been shown to release EVs which has important role in intercellular communication. In case of abnormal formation and function of the cilia causes a group of diseases including retinitis pigmentosa^48^. The data presented here provides close relationship between cilium and EVs in inherited photoreceptor degeneration in correspond to the previous observations^48^. Interestingly, cilium and EVs were colocalised explicitly which highlights the need for further detailed investigation.

EV-PARP association was studied before, especially in cancer. Some scientist observed that EVs derived from bone-marrow stromal cells were able to block reduction of full-length PARP and prevent the increase of cleaved PARP^49^. EVs were also used to carry CRISPR/Cas9 plasmids to inhibit PARP-1 expression in ovarian cancer^50^. In the RPE, it was noticed that apical released EVs carrying αB crystalin, a chaperone protein with anti-inflammatory effects, were able to reduce PARP activity in neighbouring cells^44^. When isolated EVs were analysed, it was noticed that the total number did not suffer significant changes, when untreated and treated groups were compared. Furthermore, the number of EVs expressing RPE65 was very similar in both groups. This fact makes sense, since the monolayer of pigmented cells is barely suffering any damage at early ages. However, when expression of rhodopsin – a specific protein found in rod photoreceptors – was studied, the outcome was completely different. We previously observed how PARP inhibitor olaparib was able to enhance rescue of rods (rods are the cells affected by the *rd10* mutation). Thus, it is coherent to think that a higher number of cells will produce a higher number of vesicles. On the other hand, it is remarkable that this study is the first one in observing expression of rhodopsin in EVs coming from the retina.

## Materials and Methods

### Experimental animals

*Rd10* and wild-type (*wt*) animals at P9 (for culture preparation) and P18 (*in vivo* preparations) were used irrespective of gender. The animals housed under standard white cyclic lighting and had free access to food and water. All procedures were performed in accordance with the ARVO statement for the use of animals in ophthalmic and visual research and were approved by the Tübingen University committee on animal protection (Einrichtung für Tierschutz, Tierärztlicher Dienst und Labortierkunde directed by Dr. Franz Iglauer).

### Retinal explant cultures

Serum free organotypic retinal culture method is a well established method^51^. Briefly, for the experiment, the eyes at P9 were removed aseptically and then washed with R16 medium. To remove sclera from retinal pigment epithelium (RPE), eyes were incubated in 0.12% Proteinase K (ICN Biomedicals Inc., OH, USA; 193504) for 15 min at 37 °C. Proteinase K activity was inhibited by washing step with 10% fetal calf serum (FCS). Eyes washed with R16 medium and cornea, sclera, lens and choroid were removed aseptically under the microscope (Zeiss, Stemi 2000 C). Only the retina with RPE attached remained. Eye cup cutted into four wedges and was transferred to a culture membrane insert (Millipore, Carrigtwohill, Cork, Ireland; PIHA03050) with the photoreceptor-side down. The inserts were transferred into the six well plates. Explants were incubated in R16 medium with supplements at 37 °C in a humidified 5% CO_2_ incubator between P9 from P18, the peak of degeneration. The culture medium was changed every two days and for the first two days (P9-P11) the cultures were left without treatment, and then treated for 7 days (P11-P18) with 100 nM olaparib (Selleckchem, Catalog No.S1060). Olaparib was dissolved in dimethyl sulfoxide (DMSO; Sigma; D2650) and diluted in R16 medium with supplements. The same concentrations of DMSO were added to the controls. The treatment for all animal models was administered before the degeneration peak, and culture was finished at the peak of degeneration to analyze the neuroprotective effect.

### Immunofluorescence and TUNEL assay

Animals were sacrificed at P18 with CO^2^ and their eyes enucleated. Eyecups were fixed for 1 h at room temperature (RT) in 4% paraformaldehyde (PFA) (Poysciences, Warrington PA, USA) in 0.1 M phosphate buffer (PB, pH 7.4) containing 0.2 M sucrose. After washing in PB, eyes were cryoprotected by immersion in graded sucrose (10%, 20%, and 30%) in PB. Tissues were then embedded in a tissue-freezing medium (Jung, Leica Instruments, Heidelberg, Germany). Vertical sections (12 µm) were cut on a Leica CM3050S Microtome (Leica Biosystems, Wetzlar, Germany), air dried at 37 °C for 1 h, and stored at −20 °C until use. Frozen sections from fixed tissue were air dried for 30–60 min at 37 °C.

For the immunofluorescence (IF) studies, sections were rinsed in PBS and preincubated for 1 h at RT in blocking solution containing 10% normal goat serum, 1% bovine serum albumin (BSA), and 0.1% Triton X in PBS. Primary antibodies (Rhodopsin; Merck Millipore, MAB5316, CD9; Abcam ab92726; GT335, AdipoGen AG-20B-0020) were diluted in blocking solution overnight at 4 °C. Subsequently, sections were rinsed in PBS and incubated with Alexafluor-488 conjugated secondary antibody (Invitrogen; dilution 1:250-1:750). Sections were washed in PBS and mounted in Vectashield mounting medium with DAPI (Vector, Burlingame, CA, USA).

Cell death was assessed using the terminal deoxynucleotidyl transferase dUTP nick end labeling (TUNEL) assay by means of an *in situ* cell death detection kit with fluorescein isothiocyanate as the reporter fluorochrome (Roche Diagnostics, Mannheim, Germany).

### PAR immunohistochemistry

PAR immunohistochemistry was performed with sections from rd10/corresponding wt preparations and *in vitro* rd10/corresponding wt retinae at P18. Sections were air dried 30–60 min at 37 °C and washed with PBS for 10 min. Non-specific background reduced by quenching solution which included 30% H_2_O_2_, MeOH, 0,1% PBST. After that, sections were blocked with 10% normal goat serum in 0.1% PBST for 1 h at RT and incubated with PAR antibody (PAR 10H, Alexis, dilution 1:200) for overnight at 4 °C. Biotinylated secondary antibody (Vector lab; dilution 1:150) was diluted in 5% normal goat serum in 0.1% PBST and the sections were incubated for 1 h at RT. After washing, the slides were incubated in Vectastain Elite ABC kit (Vector lab) for 1 h at RT. The color reaction was produced with DAB solution containing 20% Glucose, 0.4% NH^4^Cl, 1% Nickel ammonium sulfate, 40 mg 3,3′-diaminobenzidine (DAB), and 40 µl Glucoseoxidase. After DAB incubation, slides were washed with PB and covered by Aquatex (Merck).

### Microscopy, cell counting, and statistics

Microscopy was performed by a Zeiss Imager Z1 Apotome Microscope which has structured illumination optical sectioning and is an alternative to confocal microscopy with conventional epi-fluorescent light source. Images were taken with a Zeiss Axiocam digital camera using the Zeiss Axiovision 4.7 software. The percentages of positive cells were assessed and calculated in a blinded fashion as reported previously^9^. For each animal three fields of view at 20x magnification in central retinal areas (in proximity to the optic nerve) were analyzed and at least 3 sections next to the optic nerve were quantified for sections from *in vivo* preparations. At least 4 different animals were analyzed for each genotype. The retinal sections were collected and analyzed from different central parts of retina for organotypic retina cultures.

Values are given as mean ± standard error of the mean (SEM). Statistical analysis was performed using GraphPad Prism 4.01 software (GraphPad Software, La Jolla, CA, USA). Mann-Whitney U test was used for single comparison and One-way Anova test with Bonferroni correction was used for multiple comparisons. Levels of significance were: *p < 0.05, **p < 0.01, ***p < 0.001.

### Extracellular vesicles isolation

In brief, 1.2 mL of culture media from retinal explants at P16 and P18, treated or not, was collected and concentrated at 4 °C at 2500 × g for 2 min (Ultra-4 10 k Centrifugal Filter Devices, Amicon). EVs isolation was performed using size exclusion chromatography according to the manufacturer’s protocol for microvesicle isolation (qEVcolums, Izon Science, Oxford, UK). Fraction 8 and 9 were used for downstream applications. EV identity was confirmed by the nanoparticle tracking system NanoSight NS300 following manufacturer protocols (Malvern Instruments, Malvern, UK).

### Electron microscopy

To identity EVs typical morphology a pool of 1.5 ml of concentrated control culture media was isolated using qEVs columns. Fractions 8 and 9 were collected. A total of 3 ml of isolated microvesicles were ultracentrifugated at 150.000 × g for 90 min at 4 °C. The EVs pellet was resuspended in 30 μl PBS 1X. The sample was fixed by 2% PFA. Negative staining was performed with 2% uranyl acetate. Photomicrographs were obtained using the transmission electron microscope FEI Tecnai G2 Spirit (FEI Europe, Eindhoven, Netherlands) using a digital camera Morada (Olympus Soft Image Solutions GmbH, Münster, Germany). EVs were identified under the microscope solely based on size and morphology.

### Flow Cytometry

EVs were incubated 1 h at 4 °C in rotation with mouse anti-human CD9-APC antibody (9-A-100T, Immunostep, Salamanca), as a well-established EV marker. The sample was passed through a Gallios cytometer (Beckman Coulter, Brea, CA, USA) for 10 min. RPE65 population was detected using primary mouse-anti-RPE65 antibody (Abcam, 13826), incubated 1 hr at 4 °C in rotation, and the secondary goat anti-mouse antibody labeled with PerCP-Cy5.5 (ref: 1399990225, Immunostep) incubated for 30 min on wheel. Before the analysis, to standardize parameters, fluorescence polystyrene particles were used (SPHERO Nano Fluorescent Particle Size Standar Kit, Spherotech, Lake Forest, IL, USA). Plots were afterwards analysed with Kaluza Analysis Software (Beckman Coulter).

### Dot Blot

For the EVs protein extraction, 20 µl of RIPA buffer (Sigma-Aldrich, St. Louis, MO, USA) and protease inhibitor cocktail (Sigma-Aldrich) per 100 µl of sample were added to the EVs suspension. The samples were vortexed 6 cycles for 30 sec, sonicated using a UP200S sonicator (Hielscher Ultrasonics, Teltow, Germany) 6 cycles of 6 pulses (amplitude 30%) and stored at −20 °C until further processing. EVs protein amount was quantified by FluoroProfile Protein Quantification Kit (Sigma-Aldrich). Equal amount of protein was loaded and measured by dot blot in 0.45 µm nitrocellulose membranes. Membranes (IPVH00010, Merck Millipore) were incubated overnight at RT with antibodies against opsin (1:10,000; Sigma-Aldrich) and CD9 (1:250; Abcam, Cambridge, UK) as loading control. Lastly, membranes were incubated for 2 h at RT with a mouse anti-rabbit IgG-HRP (1:10,000; Santa Cruz Biotechnology, Dallas, TX, USA). Dots were visualized with ECL (Pierce, Thermo Scientific, Rockford, Il, USA) and detected with Image Quant LAS-400 mini (GE Healthcare, Uppsala, Sweden). Protein levels were quantified by densitometry using Quantity one (4.6.6, Biorad, Hercules, CA, USA).

## Supplementary information

Supplementary info

## Data Availability

All data generated or analysed during this study are included in this published article and its Supplementary Information File.
