# Supplementary material for: Poly ADP ribosylation and extracellular vesicle activity in rod photoreceptor degeneration
Source: Sci Rep. 2019 Mar 6;9:3758. doi: 10.1038/s41598-019-40215-3 (PMC6403254; doi:10.1038/s41598-019-40215-3)
Supplement: Supplementary file 1 — Supplementary info [file 41598_2019_40215_MOESM1_ESM.pdf]

Poly ADP ribosylation and extracellular vesicle activity in rod photoreceptor degeneration

*Lorena Vidal-Gil[2,3] ¶, Javier Sancho-Pelluz[3]¶, Eberhart Zrenner[1], Maria Oltra[3], Ayse Sahaboglu[1]**

[1]Division of Experimental Ophthalmology, Institute for Ophthalmic Research, Tuebingen, GERMANY.
[2] Escuela de doctorado, Universidad Católica de Valencia San Vicente Mártir
[3]Neurobiología y Neurofisiología, Facultad de Medicina y Odontología, Universidad Católica de Valencia San Vicente Mártir

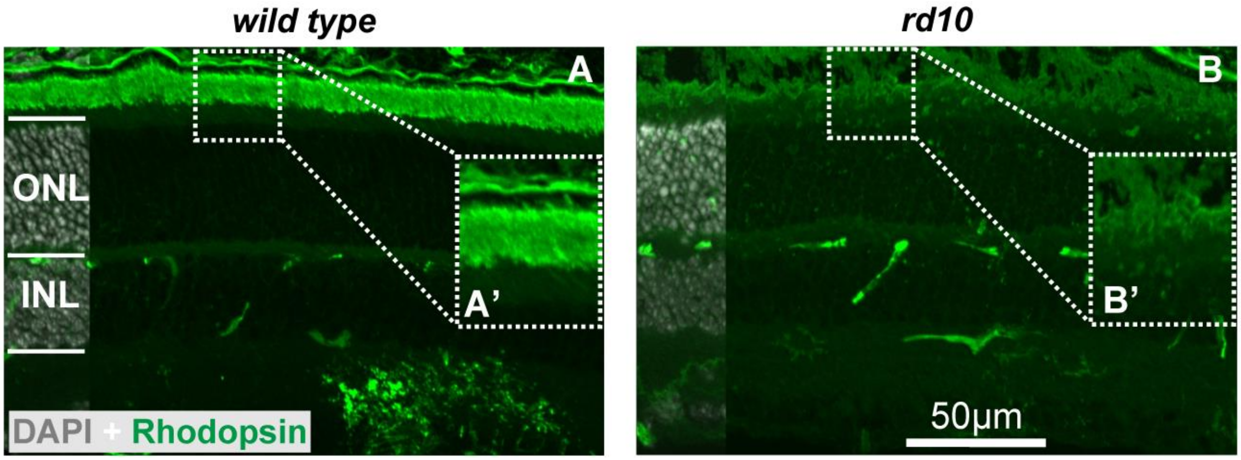

S1: Expression of rhodopsin in rd10 and wt ex vivo retinae at PN18. Immunostaining for rhodopsin showed strong immunoreactivity for wt retinae (A) although there was hardly any immunoreactivity for *rd10* mice (B).

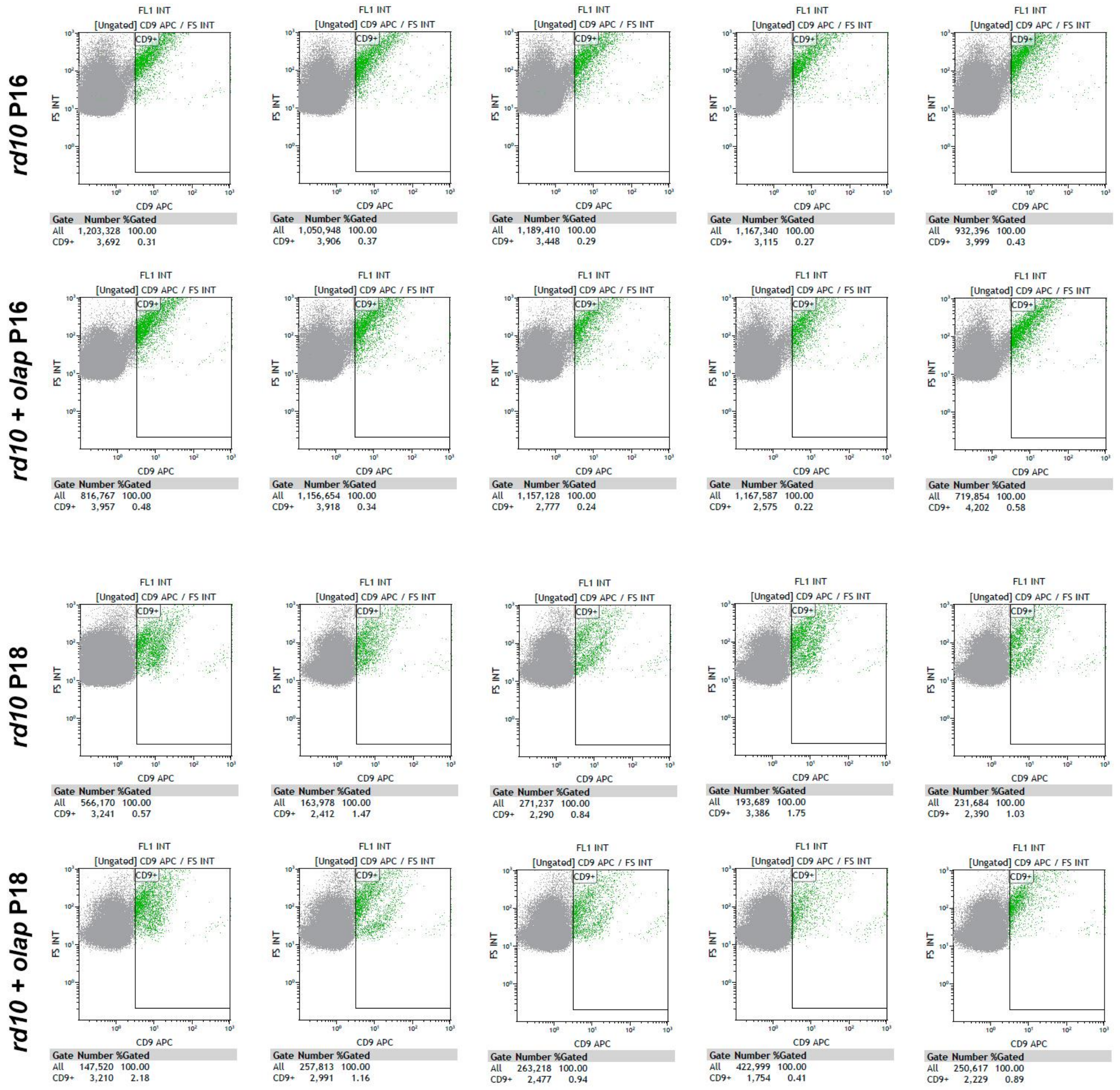

S2: Flow cytometry analyses of EVs from retinal cells expressing CD9. Flow cytometry analyses of CD9-positive exosomes released from untreated and treated (Olaparib) retinas from *rd10* mice. A representative dot plot of at least three experiments showing the percentage of co-labeled exosomes with FITC-antibodies.

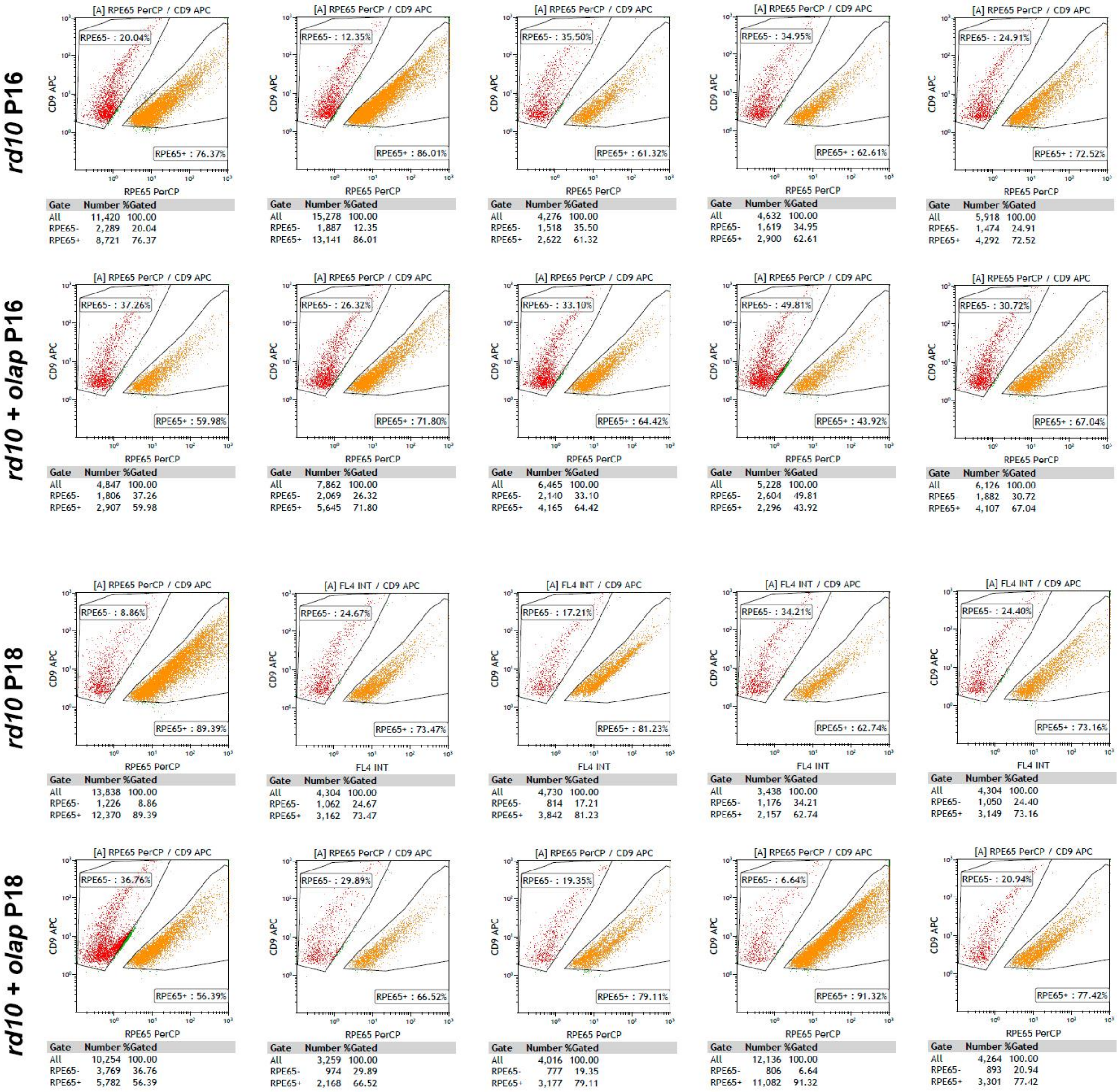

S3: Flow cytometry analyses of exosomes from retinal cells expressing RPE65. Flow cytometry analyses of RPE65 expression on CD9-positive exosomes released from untreated and treated (Olaparib) retinas from *rd10* mice. A representative dot plot of at least three experiments showing the percentage of co-labeled exosomes with FITC-antibodies and APC-conjugated CD9.
